# Diet, Oxidative Stress, and Blood Serum Nutrients in Various Types of Glaucoma: A Systematic Review

**DOI:** 10.3390/nu14071421

**Published:** 2022-03-29

**Authors:** Maryla Młynarczyk, Martyna Falkowska, Zuzanna Micun, Iwona Obuchowska, Jan Kochanowicz, Katarzyna Socha, Joanna Konopińska

**Affiliations:** 1 Department of Ophthalmology, Medical University of Białystok, M. Skłodowskiej-Curie 24a, 15-276 Białystok, Poland; mromaniuk2121@gmail.com (M.M.); z.micun@wp.pl (Z.M.); iwonaobu@wp.pl (I.O.); 2 Department of Bromatology, Faculty of Pharmacy with the Division of Laboratory Medicine, Medical University of Białystok, Mickiewicza 2D, 15-222 Białystok, Poland; martyna.falkowskaa@gmail.com (M.F.); katarzyna.socha@umb.edu.pl (K.S.); 3 Department of Neurology, Medical University of Białystok, M. Skłodowskiej-Curie 24a, 15-276 Białystok, Poland; kochanowicz@vp.pl

**Keywords:** primary open angle glaucoma, primary angle-closure glaucoma, PEX glaucoma, diet, oxidative stress, nutrients serum level

## Abstract

Glaucoma is one of the most common causes of irreversible vision loss worldwide. It is an insidious disease with a multifactorial pathogenesis. Despite progress in treatment methods, prevention and lifestyle modifications may be useful in slowing the progression of this disease. This systematic review aimed to evaluate the influence of diet, oxidative stress, and disturbances in blood serum levels of nutrients on the incidence and severity of glaucoma based on scientific reports on the role of nutrition in the pathogenesis and course of glaucoma. This paper presents an analysis of the above issues; however, further research is required to develop this topic. Future clinical trials are needed to assess the influence of nutrition and to develop nutritional management strategies for patients with glaucoma.

## 1. Introduction

Glaucoma is a multifactorial disease characterized by progressive neuropathy of the optic nerve. It affects approximately 70 million people worldwide, and its incidence is predicted to increase [1]. Changes in the morphology of the optic nerve disc lead to visual-field loss, progressive deterioration, and, finally, irreversible vision loss. Glaucomatous neuropathy is mainly caused by persistently high intraocular pressure (IOP); however, it is possible to develop glaucoma when the pressure is within the normal range (normal tension glaucoma) [NTG] [2].

Under normal conditions, the aqueous humor flows from the posterior chamber to the anterior chamber of the eye and is then drained through the outflow pathways: the trabecular meshwork (TM), the choroidal, and the iris. In primary open-angle glaucoma (POAG), outflow of the aqueous humor is impeded because of increased resistance at the levels of the above outflow pathways [3]. One of the most common causes of secondary OAG is pseudoexfoliation syndrome (PEX), characterized by the accumulation of fibrous deposits in the anterior segment of the eye, including the anterior chamber angle and lens capsule [4].

In angle-closure glaucoma (ACG), the peripheral part of the iris obscures the TM, which blocks the outflow of the aqueous humor [1]. In the course of glaucoma, morphological changes occur in many structures of the eye, including optic nerve fiber atrophy, apoptosis of retinal ganglion cells, excessive loss of the TM cells, and changes in the morphology of cells lining Schlemm’s canal (SC) [1]. The main goal of pharmacological treatment is IOP lowering; however, it is associated with adverse events and may be inconvenient for the patient. Surgical methods that significantly improve glaucoma control are constantly improving. Surgical treatment is directed at increasing the outflow of the aqueous humor using various methods, including trabeculectomy, canaloplasty, or the latest minimally invasive glaucoma surgery techniques [5]; although these are highly effective, they are not free from complications. Glaucoma is an insidious disease that develops asymptomatically for an extended period of time, during which it is often unnoticed. The changes that occur during its course lead to irreversible vision loss, often impeding patients from engaging in professional activities and significantly altering everyday functioning [6]. It is therefore of paramount importance to investigate additional therapeutic methods to prevent and limit its course. There are scientific reports on the influence of nutrition and levels of selected nutrients on the risk of glaucoma or progression of the disease. The occurrence of oxidative stress in the body is important in the pathophysiology of glaucoma. Oxidative stress results from imbalance between the production of reactive oxygen species and the capacity of antioxidants and repair systems, leading to damage to lipids, proteins, and DNA structures, and subsequently to cellular destruction [7]. Deficiencies or over-supplementation of certain macro- and micronutrients also adversely affect the course of glaucomatous neuropathy. Given the multifactorial nature of glaucoma and its chronic course, the role of nutrition and appropriate choice of dietary nutrients should be considered as an adjunct to treatment or to inhibit disease progression.

The aim of this systematic review was to present an overview of the scientific literature on nutrients (vitamins, trace elements, and other antioxidants) that are known to have an impact on the development of different types of glaucoma and to determine the association of these nutrients with the course of the disease. There are some existing reviews on the topics of nutrition and lifestyle and their relationship with the occurrence of glaucoma [8,9,10]; however, our review is additionally enriched with a description of the negative impact of high serum levels on selected nutrients and the development of glaucoma. Additionally, given the multifactorial etiology and diversity of the course of glaucoma, we focused on discussing the influence of selected components on the course of individual types of glaucoma. The review discusses POAG, NTG, ACG, and PEXG. The strength of this work is that is provides information about the influence of dietary nutrients on the occurrence or course of glaucoma and comparatively analyzes the serum levels of the relevant nutrients.

## 2. Nutrition, Blood Nutrient Levels of Oxidative Stress, and Glaucoma

In addition to elevated IOP, distressed ocular blood flow and oxidative stress are the possible concurrent risk factors that may have influence on glaucomatous optic neuropathy (GON). Glaucoma is clearly associated with high intraocular pressure or blood-flow dysregulation. Nitric oxide (NO) appears to be strictly involved in regulation of those two mechanisms. NO leads to the relaxation of the underlying vascular smooth muscle cells and vasodilation. It improves blood flow through the vessels, which may have a protective effect on the optic nerve. On the contrary, in case of an endothelial dysfunction, lack of NO production from damaged cells in a vessel may potentially result in a vasospastic reaction [11]. Trabecular meshwork (TM) cells, being smooth muscle-like cells, rapidly relax and decrease their cellular volume in response to NO. These changes account for the improvement in the TM morphology, leading to an increase in aqueous humor outflow and IOP reduction [12]. Collapse or narrowing of Schlemm’s canal (SC) leads to NO production by endothelium [13]. NO produced by SC cells at elevated IOPs can affect smooth muscles in distal vessels and thus lower IOP. In addition, NO influences intercellular junctions and plays a major role in vascular permeability and might contribute to trabecular outflow resistance [14,15]. NO is also involved in apoptosis, a mechanism of cell death that can lead to retinal ganglion cell loss in glaucoma. NO can react with the superoxide anion to form very toxic peroxynitrite that can trigger apoptosis. Increased amounts of peroxynitrite have been found in the optic nerve head of glaucoma patients. Moreover, interaction between NO and metals, such as iron or copper, may also lead to production of toxic products [11].

It was suggested that oxidative stress elaborates in the pathogenesis of glaucoma and unstable ocular blood flow may lead to the production of free oxygen radicals and oxidative damage in the retinal ganglion cells. Oxidative stress is known to upregulate the synthesis of neuronal and inducible nitric oxide synthase (NOS), leading to overproduction of NO, which acts as a neurodestructive agent by the production of peroxynitrites. During reperfusion injury, a high concentration of superoxide radicals and NO results in the formation of highly damaging peroxynitrites [16,17], which may lead to retinal ganglion cell loss and GON.

### 2.1. Primary Open-Angle Glaucoma

As the oxidative stress plays a role in the pathogenesis of glaucoma, the protective action of dietary nutrients with antioxidant or pro-oxidant effects should be considered. The effects of nutrition on the risk of POAG were evaluated in the prospective Rotterdam study with a population-based cohort of more than 3500 participants. Consumption of specific nutrients was determined by questionnaires, and the participants were free of glaucoma at the time of the study. Participants diagnosed with glaucoma consumed a diet with a lower intake of antioxidant-rich foods, such as β-carotene, retinol, and B vitamins, i.e., B1 and B12, and a higher intake of magnesium- and vitamin E-rich foods than participants without glaucoma. The difference in the incidence was higher when dietary supplement users were excluded. These results may indicate the involvement of these components as factors that increase or decrease the risk of POAG [18].

Braakhuis et al. studied the relationship between the use of various dietary products containing antioxidants and the occurrence of eye diseases related to oxidative stress, such as POAG, cataracts, or age-related macular degeneration. The occurrence of these diseases was less frequent among participants with an increased dietary supply of fruits and vegetables rich in antioxidants, vitamin C, and β-carotene [19]. Other components whose dietary deficiencies may be associated with the occurrence of glaucoma are vitamin A and vegetable oils [20].

Both deficiencies and excessively high serum levels of particular nutrients often reflect disorders in the body. Markers of oxidative stress, as a cause of glaucoma development, and serum antioxidant or oxidant levels appear to be helpful in the diagnosis and study of disease progression.

The relationship between visual-field changes in patients with POAG and serum antioxidant and oxidant levels has been studied. Using the Diacron reactive oxygen metabolites, biological antioxidant potential (BAP), and sulfhydryl tests, markers of oxidative stress (lipid peroxides, iron-reducing activity, and thiol antioxidant activity) were evaluated using a free radical analyzer. A significant association was observed between decreased BAP values and significant visual-field loss in patients with POAG. The weaker antioxidant capacity of the body may be associated with a more severe course of glaucoma, as reflected by the more extensive changes in the visual field. Additionally, the changes are more intense in POAG than in glaucoma with normal IOP [7].

Uric acid is a well-known antioxidant and a free-radical neutralizer in the body. It is a natural metabolite of purines, the main source of which is meat. The uric-acid level in the blood serum of patients with POAG was assessed and it was observed that its level was significantly lower in patients than in controls. Additionally, the participants were divided into groups according to the progression of glaucomatous changes in the visual field (mean deviation), reflecting the stage of the disease. The lowest serum uric-acid levels were noted in patients in the group with the most severe disease course; however, when the patients were divided according to sex, a statistically significant relationship was observed in the male group [21].

Disturbed blood flow regulation affects optic nerve function. NO, a vasodilator, improves blood flow through the vessels, which may have a protective effect on the optic nerve. The effects of dietary NO on the development of POAG have been previously studied. High IOP was observed to be less frequent in participants consuming a diet rich in green leafy vegetables containing large amounts of nitrogen compounds, a source of NO [22].

The involvement of iron as one of the body’s oxidants may be considered in the pathogenesis of glaucoma. According to studies, excessive dietary iron supply may correlate with an increased risk of glaucoma [20,23]. Moreover, a study by Lin et al. evaluated the relationship between the serum levels of ferritin, which is the body’s iron store, and the occurrence of glaucoma. The prevalence of glaucoma was higher among participants with high ferritin levels, which may be related to the role of oxidative stress in the pathogenesis of glaucoma [24]. Additionally, a higher prevalence of glaucoma has been reported in individuals with increased calcium and iron supplementation [23].

Coenzyme Q10 (CoQ, CoQ_10_) is a vitamin-like natural compound that plays an essential role in the electron transport chain, production of adenosine triphosphate (ATP), and inhibition of reactive oxygen species (ROS). It is a potent antioxidant and protective agent, and its effects have been shown in neurodegenerative diseases, including Leber Hereditary Optic Atrophy, Parkinson’s disease, and Huntington’s diseases [25,26]; it also has an effect on retinal ganglion cells (RGCs) [27]. In preclinical trails [28,29,30], topically administrated CoQ_10_ helped reduce the levels of extracellular glutamate, retinal damage, and cellular apoptosis. Treatment with the active form of CoQ_10_—ubiquinol—also enhances the survival rate of RGCs by decreasing apoptosis and increasing the expression of TFAM/oxidative phosphorylation (OXPHOS) complex 2 protein [25,28]. CoQ_10_ along with α-tocopherol (vitamin E) in POAG patients improved the retinal biometric responses (pattern electroretinogram improvement) and bioelectric cortical responses (visual evoked potentials improvement) [28,30,31,32,33].

### 2.2. Normal Tension Glaucoma

The effects of niacin (vitamin B3) deficiency and the development of glaucoma are considered to be independent of IOP. Decreased intake of products rich in vitamin B3 has been reported in individuals with glaucoma with pressure lower than 21 mmHg [34]. Besides the influence of individual nutrients on the occurrence of glaucoma, the nutritional status of the body seems to be an important factor modulating the risk of this disease. An association between reduced nutrient intake and low body mass index (<18.5 kg/m^2^) in women and an increased incidence of NTG in these individuals have been demonstrated [35].

Retinol belongs to the group of vitamin-A derivatives and has antioxidant function. The level of retinol in the blood serum of individuals with NTG was tested. The results were compared with those from participants with POAG and of a control group. Significantly lower levels of retinol in the serum of participants with NTG were observed compared to the control group and to participants with glaucoma with high IOP, which may indicate the influence of the retinol level on the occurrence and course of NTG [36].

Other compounds with a potential influence on the course of NTG are anthocyanins and *Ginkgo biloba* extract [37].

There are reports that Ginkgo biloba may have a positive impact on vascular circulation and oxidative stress. Both mechanisms are known to be involved in pathogenesis of glaucoma. Ginkgo is known to improve ocular blood flow. Study by Chung et al. [38] evaluated a possible therapeutic effect of Ginkgo biloba extract (BGE) and found that BGE significantly increased end diastolic velocity (EDV) in the ophthalmic artery (OA) with no side effects or alterations in arterial blood pressure, heart rate, or IOP. These data support the hypothesis that Ginkgo may be beneficial for glaucomatous disease where ocular microcirculation is impaired [39]. Zhang et al. investigated the therapeutic effect of BGE on cerebral microcirculation hypertension and concluded that it can be used to regulate hypertension and to protect the cerebral microcirculatory function. Although the investigation was conducted at the cerebral level, it may be assumed that a similar conclusion can be drawn for the eye [40].

Glaucoma patients tend to suffer from ischemia-reperfusion injury. Conclusions based on the results observed in rat diabetic retina and ischemia-reperfusion in rat heart allow to consider similar beneficial effects of Ginkgo in the human optic nerve head [41]. Normal tension glaucoma is particularly affected by optic disc hemorrhages which are associated with a higher risk for progression [42], but there is no treatment available at present to reduce this [43]. It is known that GBE can prevent vasospasm in subarachnoidal hemorrhages; therefore, it can be assumed that Ginkgo also has a beneficial effect in glaucoma patients with optic disc hemorrhages.

Mitochondria play a major role in neurodegenerative diseases, including glaucoma. Ginkgo contains many compounds, such as, e.g., flavonoids, and vitamins E and C, which have been proven to exert antioxidative properties by delivering electrons to free radicals [44]. An in vitro study by Eckert et al. supports neuroprotective properties of Ginkgo extract and its antiapoptotic abilities at the mitochondrial level [45].

Ginkgo biloba extract therapy could be beneficial for glaucoma, but it should be recommended only as an adjuvant treatment for normal tension glaucoma patients or in cases with high tension glaucoma progressing despite a normalized IOP. Pathogenesis of these conditions is strictly connected with microcirculation and oxidative stress disturbances and so they might be potentially treatable with Ginkgo. There is currently no alternative treatment for such cases. Moreover, significant positive effect in favor of Ginkgo was observed both in experimental glaucoma animals and in glaucoma patients and no single publication contradicts the positive effect of Ginkgo [43].

These are known to improve blood circulation and have a protective and antioxidant effect on cells. The effects of anthocyanin therapy or *Ginkgo biloba* extract therapy and their influence on the improvement of vision parameters in individuals with NTG were analyzed. Improvement in visual acuity (logMAR best-corrected visual acuity) and visual-field parameters (Humphrey Visual Field test) was observed in connection with the administration of anthocyanins and improvement in visual-field parameters after *Ginkgo biloba* extract therapy. These results may suggest a positive influence of these compounds on the course of NTG [46,47]. However, these findings were not confirmed in the study by Guo et al. [48]. Other studies confirmed the IOP lowering effect of anthocyanins in healthy subjects [49].

Omega-3 polyunsaturated fatty acids (PUFAs) are essential nutrients that occur as long-chain acids (decosahexaenoic acid (DHA), eicosapentaenoic acid (EPA)) and short-chain acids (α-linolenic acid (ALA)) [50]. As it was shown in Bazan’s study, DHA is concentrated in the retina and synaptic membranes and plays a significant role in rhodopsin regeneration [51]. Omega-3 PUFAs also show anti-inflammatory action, meaning they can be used for treating chronic inflammatory diseases, such as glaucoma [52,53]. Regarding PEX glaucoma, a 6-month open-label randomized trial showed that oral DHA supplementation ameliorated IOP in patients in the experimental group [54]. A similar conclusion was presented in normotensive adults [55]. A very low ratio of high-tension glaucoma (HTG) to NTG in Japanese people (8% of glaucoma patients have HTG) and the high ratio in Japanese Americans (17% of glaucoma patients have HTG) suggest that factors other than genetic factors, such as a diet high in omega-3 products, can play a role in decreasing the IOP [56].

Studies have shown that some calcium channel blockers have a positive effect on ocular blood flow and visual field in NTG. However, systemic side effects such as hypotension and bradycardia limit the use of calcium channel blockers for the treatment of glaucoma [57]. Nevertheless, Mg acts as a natural physiologic calcium channel antagonist with minimal cardiovascular side effects and tends to increase blood flow and decreases vascular resistance within the vascular beds. Elevated levels of Mg inhibit ET-1 induced contraction in porcine ciliary arteries and may regulate the perfusion abnormality at microcirculatory level. Therefore, Mg supplementation may have a therapeutic effect by decreasing the ocular vascular tonus via inhibition of the ET-1 induced contraction. Aydin et al. investigated the efficacy of oral Mg therapy on visual field perimetry indices and ocular blood flow in only NTG patients in a prospective controlled randomized clinical trial. Fifteen NTG patients received 300 mg oral Mg for one month and blood flow velocity of orbital vessels such as ophthalmic, posterior ciliary, and central retinal arteries was measured by color Doppler imaging. After one month, the improvements in visual field mean deviation and pattern standard deviation were found statistically significant in the study group compared to control group [57].

An association between reduced nutrient intake and low body mass index with an increased incidence of NTG in women has been mentioned. Low BMI is a major feature of Flammer syndrome (FS) which is characterized by general symptoms such as low body mass index, cold extremities combined with slightly increased core temperature, prolonged sleep onset time, reduced feelings of thirst, increased sensitivity to smell and also for certain drugs, and increased retinal venous pressure, as well as ocular comorbidities, i.e., normal-tension glaucoma, anterior ischemic optic neuropathy, retinal vein occlusions, Susac syndrome, and central serous chorioretinopathy [58]. In these patients, the plasma level of endothelin-1 is increased, and the gene expression in lymphocytes is changed. In the eye, the retinal vessels are rigrid and their longitudinal variability is greater; moreover, the autoregulation of ocular blood flow is decreased. Glaucoma patients with FS have an increased frequency of the following: optic disc hemorrhages, activated retinal astrocytes, elevated retinal venous pressure, optic nerve compartmentalization, fluctuating diffuse visual field defects, and elevated oxidative stress. Study by Konieczka et al. conducted on 246 normal tension glaucoma patients and 1116 control subjects revealed that there is an association between NTG and FS. If future studies confirm this relationship, treatment of FS may help to prevent NTG or to slow down its progression, which eventually may lead to more efficient and more personalized treatment [59].

### 2.3. Pseudoexfoliation Glaucoma

Some reports have suggested an effect of homocysteine on the deposition of PEX material and the occurrence of pseudoexfoliation glaucoma (PXG) [60].

An excess of methionine, mainly from animal proteins, combined with a simultaneous deficiency of vitamin B6, B12, and folate lead to an increase in the level of homocysteine. Previous studies have shown that increased intake of total folate was associated with a reduction in the risk of glaucoma; however, an increased effect was observed when folate was obtained from additional supplementation, besides the diet [61].

Additionally, consumption of dietary products with high folate content, which is known to lower homocysteine levels, reduces the risk of PXG [62]. Disturbances in the oxidative balance of the body have also been linked to the occurrence of glaucoma in the course of PXG. Serum samples were examined using spectrophotometric and enzymatic methods, and the total antioxidant status (TAS) was assessed in individuals with PEX glaucoma. Reduced levels of antioxidants were observed in the serum samples of patients compared to those of controls, which may indicate the involvement of oxidative stress in the pathogenesis of PXG [63].

Many trace elements affect cell and tissue functions. Reports have focused on the serum levels of trace metals and toxic elements in relation to glaucoma. Elevated levels of elements such as manganese (Mn), molybdenum (Mo), and mercury (Hg) have been observed in the serum of individuals with PXG, which may be related to the role of these elements in the pathogenesis of this condition [64].

### 2.4. Angle-Closure Glaucoma

Similar findings have been reported for ACG. In a study by Li et al., both TAS and superoxide dismutase levels were decreased, while the levels of oxidative compounds such as malondialdehyde (MDA) were increased in participants with advanced ACG with extensive visual-field loss, which may be a prognostic or pathogenetic factor in ACG [65].

Similar observations were reported in a study of patients with primary ACG regarding the uric-acid levels. In these patients, serum uric-acid levels were also lower than those in the control group, and the disease course was most severe in these patients [66].

## 3. Glaucoma and Vitamins

Supplementation with specific vitamins is recommended in the course of many diseases. The relationship between the intake of antioxidant vitamins A, C, and E, their serum levels, and the incidence of glaucoma was studied. No correlation was observed between the serum levels of vitamins A, E, and C and the occurrence of glaucoma. However, less frequent occurrence of glaucoma was found in participants who received supplementation with high doses of vitamin C [67].

In a study by Zanon-Moreno et al., the influence of the polymorphism of some genes related to vitamin C and E on the risk of POAG was investigated. A relationship between the rs1279683 polymorphism of the vitamin C cotransporter gene and the rs737723 polymorphism of the vitamin E-related protein gene and increased incidence of glaucoma was observed. Additionally, lower serum levels of vitamin C and E were noted in individuals with glaucoma [68].

Similar results have been reported for vitamin D in its form 25 (OH). Neither low nor high serum levels of this vitamin are associated with changes in IOP. Additionally, in individuals with low serum vitamin D levels, no changes in IOP were observed after supplementation with 20,000 IU of vitamin D twice weekly [69]. Moreover, in a study by Kim et al., glaucomatous changes were observed more often in women with low vitamin D 25 (OH) serum levels [70].

In another study, the serum level of vitamin 25 (OH) D3 was determined, and the relationship between the polymorphism of some genes encoding vitamin D3 receptors and the occurrence of POAG was investigated. A lower serum level of vitamin D3 was found in participants with POAG. Additionally, the presence of the vitamin D3 receptor Bsml and Taql genotypes was associated with a higher incidence of POAG [71].

As mentioned earlier, the relationship between homocysteine levels and the occurrence of PEX glaucoma is known, as is the protective effect of folate (vitamin B9). The relationship between serum levels of B vitamins, i.e., vitamins B6, B12, and folate, and the level of homocysteine in various types of glaucoma (POAG, PXG, and NTG) was investigated. A high level of vitamin B6 in NTG and POAG was observed compared with the control groups and an increased level of homocysteine in PXG, but no statistically significant differences between the level of homocysteine and other types of glaucoma were shown [72]. Similarly, in a study by Kang et al., the effects of dietary supplementation with B vitamins B6, B9 (folic acid), and B12 were studied. No association was found between the occurrence of PXG and diets rich in vitamins B6 and B12 [62].

## 4. Discussion

The information contained in our analysis strongly indicates that there is a connection between diet and disturbances in the levels of selected blood serum elements and the occurrence of different types of glaucoma. These factors may also influence disease progression. The influence of oxidative stress and the antioxidant capacity of the body on the occurrence of glaucoma is particularly marked. In a study by Mousa et al., TAS was evaluated in serum samples from patients with POAG, ACG, and PXG. The involvement of oxidative stress in the pathogenesis of glaucoma is supported by the fact that reduced serum TAS levels were found in all aforementioned types of glaucoma [73].

In addition, reduced antioxidant levels can be considered when assessing the severity of the course of POAG, as indicated by a study by Abu-Amero et al. [74]. Moreover, disorders in the oxidative balance, which includes increased levels of selected oxidants, occur in PEX and consequently in PXG. Under these conditions, increased levels of MDA, which is involved in lipid oxidation, were observed. Simultaneously, the levels of antioxidant enzymes decrease [75].

The results of the analyzed studies suggest a protective effect of a diet rich in antioxidants and oxidative imbalance as a possible pathogenetic factor in different types of glaucoma. However, our study had some limitations. The studies included in the analysis differed in terms of inclusion criteria and number of participants. In addition, the definition of glaucoma was not precisely specified in some articles, which could have affected the results, especially concerning the division into different types of glaucoma. Some studies included questionnaires in the methodology that were completed subjectively by the participants, which could have led to discrepancies in the results obtained. A meta-analysis that considers the division into glaucoma types, assessment of the body’s oxidative balance, and evaluation of the diet and eating habits of the participants included in the study is needed to explore the topic more thoroughly.

Further, our study did not consider other aspects relating to the onset of glaucoma. Indeed, many etiological factors are involved in the pathophysiology of this disease. In addition to genetic causes, environmental influences, such as pesticide exposure, lifestyle, and dietary patterns, also play a role. Finally, insufficient intake of dietary products rich in omega-3 fatty acids, such as fish, and heavy smoking may increase the risk of POAG [76]; however, some studies showed contrasting results [77,78].

## 5. Conclusions

Existing reports allow for a better understanding of the multifactorial etiology of glaucoma and the pathophysiological mechanisms involved in its development. Increasing the knowledge on diet, oxidative balance, and appropriate levels of particular nutrients in the blood serum may prove helpful in monitoring or diagnosing glaucoma or constitute an element of supplementary treatment for this disease. There are promising findings on the influence of certain groups of nutrients; however, their clinical validation is difficult because their administration should be applied for the entire duration of a patient’s life. Studies with longer follow ups and larger sample sizes are required to increase our understanding of the role of nutrition in glaucoma. In general, the most crucial for glaucoma patients is to follow-up with medical therapy, physician’s recommendations, and keep up a healthy lifestyle, i.e., avoid smoking, perform moderate exercise, and have a diet high in fruits and vegetables. Depending on the kind of glaucoma, the patient may support the therapy with supplying nutrients most beneficial for them.

## 6. Method of Literature Search

This systematic review followed the Preferred Reporting Items for Systematic Reviews and Meta-Analyses (PRISMA) guidelines. The study protocol is registered at the International Prospective Register of Systematic Reviews PROSPERO, supported by the Centre for Reviews and Dissemination of the University of York, under reference ID: CRD42022311445. The PubMed database was used for the literature search for articles published between November 2011 and November 2021. The terms “Glaucoma and nutrition”, “Glaucoma and nutrients”, “nutrients serum level in glaucoma”, “trace elements and glaucoma”, “diet and glaucoma”, “pseudoexfoliation glaucoma and diet”, and “angle-closure and diet” were used.

Articles inconsistent with the topic and abstracts available only as conference papers were excluded. Full-text publications were selected based on the compatibility of abstracts with the topic of the study. Additionally, the literature included in the selected articles was also reviewed to locate articles not found in the original searches.

We included prospective randomized control trials, case-control studies, cross-sectional studies, and publications with patients with a diagnosis of POAG, ACG, or PXG. Review articles and case reports were excluded.

### 6.1. Risk of Bias Assessment

The methodological quality of the included studies was evaluated independently by two authors (Joanna Konopińska and M.M.).

### 6.2. Data Extraction

**Figure 1:**
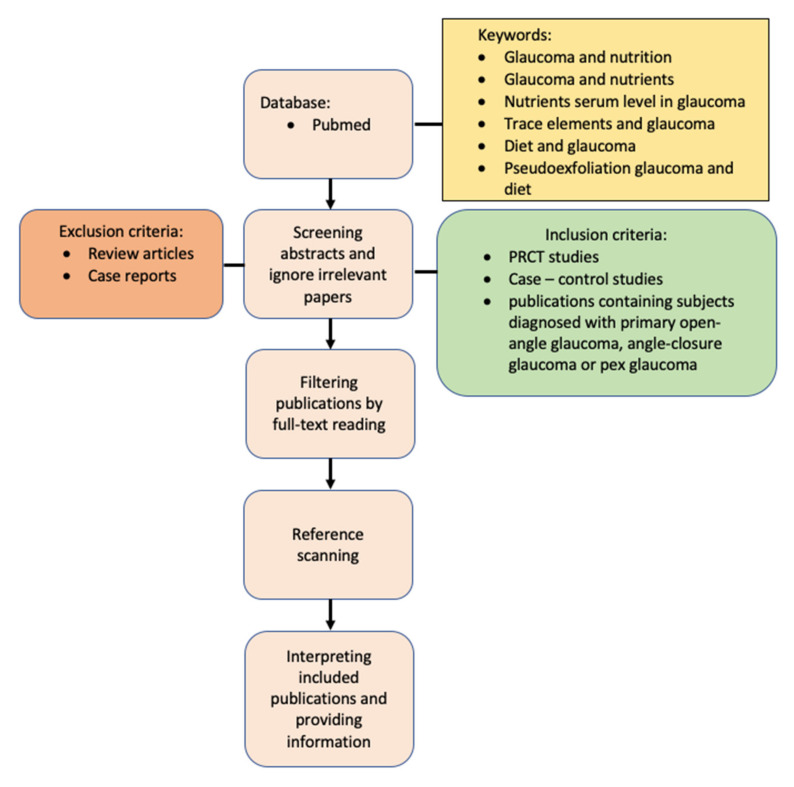
Flowchart showing proposed approach.

The demographics, participant characteristics, interventions used, and outcomes used for the purpose of this study were reviewed by the authors, and disagreements were resolved through discussion. A PRISMA schematic is shown in Figure 1. Overall, 394 publications were analyzed for diet, oxidative stress, and serum levels of selected elements in relation to the incidence of POAG, primary closed-angle glaucoma, and PEX glaucoma. From the retrieved articles, titles and abstracts were skimmed, and a thorough evaluation of the full text of suitable studies was performed. After removing duplicates, review articles, case reports, and off-topic publications, and after including publications selected during the literature review, 78 articles were included. These publications described selected diet and nutrient intake (Table 1) and changes in the serum levels of selected elements, markers of oxidative stress, and their association with glaucoma (Table 2). The remaining publications approximated the subject matter of the above analysis. In addition, the reference lists of all identified studies were reviewed and searched.

## Figures and Tables

**Table 1:** Selected diet components intake and risk of glaucoma occurrence.

References	Examined Factor	Risk of Glaucoma Occurrence
[18]	Low antioxidant intake	↑
[19]	High antioxidants intake	↓
[20]	Low vitamin A and vegetable oil intake	↑
[20,23]	High iron intake	↑
[34]	Low vitamin B3 intake	↑
[35]	Low BMI in woman	↑
[22]	High green leaves (source of NO) intake	↓
[62]	High vitamin B11 (folic acid) intake	↓
[76]	Low omega-3 fats intake	↑
[47]	Anthocyanins and Ginkgo biloba extract administration	Not mentioned, but improvement of visual function

BMI, body mass index; NO, nitric oxide; ↑ increased risk of glaucoma, ↓ lower risk of glaucoma.

**Table 2:** Serum elements and oxidative stress in glaucoma.

References	Examined Factor	Observation	Risk of Glaucoma Occurrence
[7]	Low BAP	Defects in the visual field	↑
[60]	High homocysteine level	PEX material deposition	↑
[63]	Low TAS	PEX material deposition	↑
[65]	Low TAS	Angle closure glaucoma	↑
[24]	High serum ferritin level	Increased iron resources	↑
[21]	Low serum uric acid level	Defects in the visual field	↑
[66]	Low serum uric acid level	Angle closure glaucoma	↑
[67]	Serum vitamins A, E, C level	No observation	No impact
[69]	Serum vitamin D 25 (OH) level	No observation	No impact
[64]	Higher serum level of molybdenum, manganese, mercury	PEX material deposition	↑
[75]	Higher level of MDA	Higher oxidant level	↑
[36]	Lower level of retinol	Normal tension glaucoma	↑
[68]	Polymorphism in vitamin C and E	Lower level of vitamin C	↑
[70]	Lower vitamin D 25 (OH) level	Glaucomatous changes	↑
[71]	Polymorphism in vitamin D3	Lower level of vitamin D	↑
[72]	NTG and POAG occurrence	Higher level of vitamin B6	↑

BAP, biological antioxidant potential; TAS, total antioxidant status; PEX, pseudoexfoliation syndrome; (OH), hydroxy; MDA, malondialdehyde; ↑ increased risk of glaucoma.

## Data Availability

All materials and information will be available upon an e-mail request to the corresponding author.
